# Preliminary Evaluation of the FETASS Training for Parents of Children With Autism Spectrum Disorder: A Pilot Study

**DOI:** 10.3389/fpsyg.2021.604851

**Published:** 2021-04-30

**Authors:** Bettina Brehm, Judith Schill, Reinhold Rauh, Christian Fleischhaker, Monica Biscaldi

**Affiliations:** Department of Child and Adolescent Psychiatry, Psychotherapy and Psychosomatics, Medical Center – University of Freiburg, Faculty of Medicine, University of Freiburg, Freiburg, Germany

**Keywords:** parent training, Autism Spectrum Disorder, Freiburg Parent Training for Autism Spectrum Disorder, quality of life, preliminary evaluation, parental stress, children

## Abstract

While several recent evaluation studies have shown the efficacy of parent training programs for children with neurodevelopmental disorders, manual-based training in German is still scarce. To address this gap, we developed a specific modularized training program for parents of children from preschool to pre-adolescent age with Autism Spectrum Disorder (FETASS). The overarching purpose of the FETASS intervention is to enhance social communication behavior and quality of life of the child by coaching parents. As a proximal target, the FETASS training aims to provide families with behavior management and communication strategies. The development of the training was influenced by published behavioral parent trainings and autism-specific interventions. The training comprises eight weekly sessions and targets families whose children have a diagnosis of Autism Spectrum Disorder (ASD) without intellectual and language impairments. As a preliminary pilot study, the purpose was to evaluate the acceptability of the training. Furthermore, the study aimed at initially evaluating social communication behavior, quality of life of the child, parental stress level, and parenting after training in comparison to a treatment as usual (TAU) group. Exploratively, long-term effects were investigated after 6 months of training as well. In total, 57 families participated (*n*[TAU] = 29, *n*[FETASS] = 28). Questionnaires about social communication behavior and quality of life of the child, parental stress, and parenting were administered at three time points (t1: baseline TAU/FETASS, t2: post TAU/FETASS; and t3: 6-month follow-up after FETASS). Primary outcome measures were the social communication behavior of the child and the parent’s proxy report on quality of life of the child. Secondary outcome measures were changes in parental stress and parenting behavior. Acceptability of the training was very high and we had almost no dropouts during training. Results for the primary outcome measure of social communication behavior, overall quality of life of the child, and long-term effects on social communication behavior were not significant. While long-term findings for parent stress reduction and for the quality of life of the child are promising, further research has to be done in a future randomized controlled trial.

## Introduction

Autism Spectrum Disorder (ASD) is known as a neurodevelopmental disorder with impairments in social interaction and communication skills accompanied by restricted interests, preoccupations, or stereotyped rigid behavior. Furthermore, children with ASD represent a very heterogeneous group with a large range of functional levels and varying levels of impairment, as well as varying levels of non-impairments in the different domains of development. This fact is taken into account in the diagnostic criteria of the DSM-5 [American Psychiatric Association (APA), 2013], which makes it possible to differentiate comorbidities (e.g., with or without speech delay, with or without cognitive impairment, and with or without ADHD) and better address the individual needs of each child with ASD.

Even if a child with ASD does not have additional language or cognitive impairments, families often report difficulty with everyday social situations in areas such as social communication and interaction or because of co-occurring behaviors that challenge (Brookman-Frazee et al., 2006; Lecavalier et al., 2006). It is further known that these families report on restrictions to their quality of life (Vasilopoulou and Nisbet, 2016) and a higher level of stress (Baker-Ericzén et al., 2005; Hayes and Watson, 2013). Estes et al. (2009) emphasized that a parent’s ability to manage their children’s challenging behaviors is a critical target for interventions to address the child’s functioning and decrease parental stress.

There is a substantial body of evidence that parental training can be effective to enhance the developmental trajectory of children with behavioral concerns (Webster-Stratton et al., 1989; Sanders et al., 2006; Weisz and Kazdin, 2010; Lee et al., 2012). As for parent-centered interventions in ASD, there has been an abundance of research on the efficacy of interventions such as Applied Behavior Analysis Approaches (for a review, see Virués-Ortega, 2010) or TEACCH (Mesibov et al., 2002; Turner-Brown et al., 2019) in which parents are involved as co-therapists. Furthermore, there are suggestions that behaviorally-oriented parent training is effective in reducing overreactivity in children with ASD (Matson et al., 2009; Whittingham et al., 2009). The efficacy of specific parent-mediated interventions for children with ASD is reviewed by Oono et al. (2013) and evidence for positive changes in patterns of parent-child interaction regarding shared attention is reported.

Rigorous randomized controlled trials (RCT) were conducted on the parent-led intervention “Preschool Autism Communication Therapy” (PACT; Green et al., 2010; Pickles et al., 2016). Herein, the parents with autistic children in the age range of 24–60 months are instructed to implement regular communication interventions at home to achieve improvements in child communicative behavior. Results of the RCT by Green et al. (2010) showed no immediate post-training effects on the ASD symptoms measured by the Calibrated Severity Score of the ADOS (CSS; Gotham et al., 2009). Yet, effects on proximal aspects of the dyadic parent-child interaction, e.g., “parental synchronous response to the child” could be found. Finally, these children showed long-term specific improvements of ASD symptoms in the follow-up evaluation 6 years after intervention (Pickles et al., 2016).

But overall, some quality concerns have been recently raised in the project Autism Intervention Meta-Analysis (AIM) about studies investigating efficacy of autism intervention in general, and behavioral intervention in particular (Sandbank et al., 2020; Crank et al., 2021).

As mentioned above, it is well-documented that parents of children with ASD show a higher level of stress (Davis and Carter, 2008; Estes et al., 2009; Hayes and Watson, 2013) and there is some evidence of a relationship between parent stress level and social affect and repetitive or restrictive behavior of the children (Harrop et al., 2016; Schutte et al., 2018). There is growing literature that dysfunctional parent-child interaction and parental stress can have a negative impact on the development of the autistic child (Crowell et al., 2019).

Accordingly, there have been efforts in international research to develop specific educational group training programs for families of children with ASD (Brereton and Tonge, 2005; Ingersoll and Dvortcsak, 2006; Chiang, 2013; Cutress and Muncer, 2013; Farmer and Reupert, 2013; Ji et al., 2014; Bearss et al., 2015; Ilg et al., 2016; Iida et al., 2018; Edwards et al., 2019). Even so, Preece and Trajkovski (2017) show that, in spite of the positive effect of parent education, only a few parental education group interventions exist.

For German-speaking countries, up to the last decade, there was a lack of manualized parent training programs for children from preschool to preadolescent age with ASD, especially for children without cognitive or speech impairment. To fill this gap, several groups developed parent training manuals. The TASK program (Fröhlich et al., 2014) addresses parents of young children from 3 to 6 years and teaches parents how to exercise communication strategies with their children. The FAUT-E (Schlitt et al., 2015) targets psychoeducation, behavioral family management, and communication strategies for parents of autistic children (from preschool-age to adolescence) with or without cognitive or speech impairment. At the same time and independently, the FETASS parent training has been established at our department of child and adolescent psychiatry. The intervention has been tailored to existing clinical process organization. It is suitable and feasible for the needs of families seeking specific intervention in our outpatient clinic and addresses children in the age range from preschool to pre-adolescence also focusing on Theory of Mind (i.e., understanding others’ intentions, desires, beliefs, perceptions, and emotions, for example, in tasks of false belief or of recognizing facial expressions) and on management of critical situations (e.g., changes in setting or challenging social situations). The manualized program [FETASS: Freiburger Elterntraining für Autismus-Spektrum-Störungen (Freiburg Parent Training for ASD); Brehm et al., 2015] is based on behavioral methods that take into account parental concerns regarding the upbringing of a child with an ASD.

The overarching purpose of the FETASS intervention is to enhance the social communication behavior and quality of life of the child by coaching parents. As a proximal target, the intervention aims to improve the parent-child relationship by increasing the parents’ understanding of the child as well as to teach behavior management that takes into account the special features of the child with autism, i.e., by providing a highly organized environment. Furthermore, teaching strategies for clear family communication and e.g., exercising “Theory of Mind” abilities should enhance social skills in the child. As an important mechanism of change, we assume a family process perspective (Patterson, 1982), in the sense that an adaption of parenting is supposed to have an important impact on the child’s social development and social-communicative behavior (see, e.g., Cox and Paley, 2003).

The present study is a “Phase-Two Evaluation” according to Smith et al. (2007) and aims to evaluate the acceptability of the FETASS training as a group intervention for parents of children with ASD without severe intellectual or language impairments in the age range from preschool to pre-adolescence. Furthermore, preliminary effects on social communication behavior, quality of life of the child, parental stress level, and parenting in comparison to treatment as usual (TAU) group were investigated. The hypotheses are (i) that there is a high acceptance of the training with a low dropout rate, (ii) that the training has positive effects on the social communication behavior and the quality of life of the child compared to TAU group, and (iii) that these effects persist reliably after the intervention. In this context, the TAU condition means routine clinical management in the outpatient unit (e.g., counseling, monitoring of medication and child’s development). In addition to the primary outcome measures, parenting behavior and parental stress were investigated after the training.

## Materials and Methods

### Participants and Intervention

The clinical study was conducted in an outpatient clinic of the Department of Child and Adolescent Psychiatry, Psychotherapy and Psychosomatics of the Medical Center of the University of Freiburg, Germany. The participants were parents/primary caretakers of a child between 4 and 15 years of age with a diagnosis of an ASD.

Our inclusion criteria were the following:

- Confirmed diagnosis of ASD (ICD-10: F84.0, F84.1, and F84.5) by an experienced clinician based on the “gold standard” instruments Autism Diagnostic Observation Schedule (ADOS-R; Lord et al., 1999; Rühl et al., 2004) and the Autism Diagnostic Interview-Revised (ADI-R; Lord et al., 1994; Bölte et al., 2006).
- Children without severe accompanying language impairment and without severe accompanying intellectual impairment.
- Children between preschool and pre-adolescent (mental) age.
- Full command of the German language.

Seventy-one families agreed to participate in the study. In the TAU group, one family dropped out because of a long-distance commute. Eight families in the FETASS group did not return the questionnaires after participating in the training.

**Figure 1:**
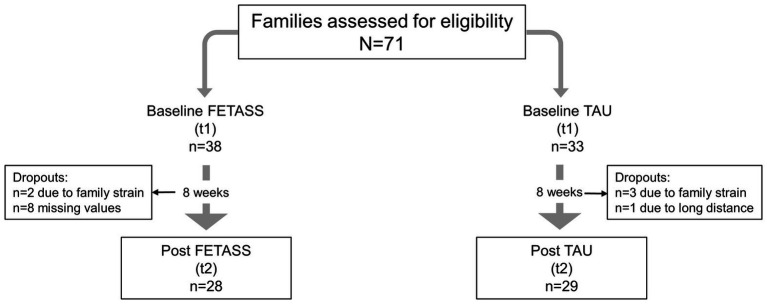
Flow of participants.

In the end, the data of 57 families (29 in the TAU and 28 in the intervention group) were included in the statistical analyses (see Figure 1). Each family was asked to nominate a primary participating parent who would complete the training and answer the questionnaires. Sixteen families were also asked to complete the questionnaires 6 months after having finished the FETASS intervention.

As is shown in Table 1, most of the children of the participating parents were male and most of them met the ICD-10 diagnostic criteria for Asperger Syndrome.

**Table 1:** Sample characteristics with gender and diagnoses of the children.

	TAU		FETASS	
	*n*	%	*n*	%
Child’s gender				
Male	23	79.3	24	85.7
Female	6	20.7	4	14.3
Diagnoses				
Asperger Syndrome (ICD-10: F84.5)	14	48.3	15	53.6
Childhood Autism (ICD-10: F84.0)	8	2.6	5	17.9
Atypical Autism (ICD-10: F84.1)	7	24.2	8	28.6

TAU, treatment as usual; FETASS, Freiburg Parent Training for ASD.

All regular participants in the FETASS intervention group were mothers, six fathers out of 28 (21.4%) of the intervention group participated regularly as well.

In our sample, 48 of 57 (84.2%) had one child with ASD in the family, 9 of 57 (15.8%) had two children with ASD. None of the families had more than two children with ASD.

The FETASS program consists of eight weekly sessions. Small groups of up to eight parents are led by two therapists. Practical exercises, working in small groups, and discussion are carried out with the help of a workbook and presentation slides. During the FETASS intervention, parents work on individual goals. After every session, parents are asked to do homework. In sessions 1 and 2, the parents receive information about special features and explanatory models of ASD, especially with reference to Theory of Mind. The next step is promoting a good relationship with the child and perceiving the strengths of the child. The parents set individual child-centered goals they want to focus on during the training. In session 3, parents are taught to provide their child with visualizations for routines at home according to TEACCH principles (Mesibov et al., 2002; e.g., schedules, prompting strategies, visualizations, balance between demand and low arousal). Positive and negative reinforcement strategies like implementing token systems, negative consequences, or extinction are taught in sessions 4 and 5. Session 6 comprises communication strategies, i. e. promoting explicit and clear communication, prompting social situations, e.g., asking for help, or supporting the child in understanding other minds. Session 7 aims to identify and prepare critical situations. In the last session, parents are taught how to understand and manage autism-related special behavior (social interaction, restrictive interests, high repetitive activities, and sensory difficulties) and challenging situations (for an overview of the content, see also Supplementary Material).

### Procedure

The study was approved by the Ethics Committee of the University of Freiburg (approval number: 382/14-Evaluation of the FETASS training for parents of children with Autism Spectrum Disorders) and was registered in the Deutsches Register für Klinische Studien (DRKS; DRKS-ID: DRKS00009761).

The present study was a self-financed, non-randomized clinical study with an intervention group and a TAU group. The parents either received the immediate intervention (intervention group) or TAU for 8 weeks. After the 8 weeks of TAU, the TAU group received the FETASS training for ethical reasons. The training was delivered by two therapists who were regularly supervised by one of the authors.

Parents received the questionnaires during one supplemental appointment 8 weeks before the training began. During the TAU period, the children could still be treated by a child and adolescent psychiatrist with different numbers of appointments and/or interventions (e.g., medication or other interventions). The allocation to intervention condition (FETASS vs. TAU) was not randomized, but decided according to the order of registration. We tested feasibility in terms of recruitment and retention/dropout rate.

In addition, we exploratively asked almost a third of the participants (*n* = 16) either of the FETASS or of the TAU group to fill out the questionnaires again 6 months after the training for an exploratory follow-up investigation (t3). There are no significant differences of the follow-up sample with respect to baseline characteristics of age, IQ, SRS-T-Total, QL-Total-LQ0-28, ESF-PS, and EFB-K-Total.

Since this was a pilot study, a sample size calculation was not performed (Eldridge et al., 2016). In order to get an accurate estimate of the SD of the outcome measure for the main trial, we followed the recommendations of Whitehead et al. (2016) who proposed sample sizes of 25 per intervention arm for small standardized effect sizes (*d* = 0.2) for a main trial designed with 90% power and two-sided 5% significance. Therefore, we aimed to get a pilot trial total sample size of about *N* = 50 participants.

Although in the literature, the effects of other parent training are frequently reported as medium or large (for ADHD: *d* = 0.56–0.86; see Weisz et al., 1995; Serketich and Dumas, 1996; for Triple P Stepping Stones: medium to large; see Tellegen and Sanders, 2014), we based our sample size justification on a small to medium effect, because at the beginning of our study, no effect sizes for training programs for parents with children on the Autism Spectrum have been reported.

### Materials

The parents were administered the following questionnaires for evaluation:

In the Social Responsiveness Scale (SRS; Constantino and Gruber, 2005; German translation: SRS; Bölte and Poustka, 2008), the parents rate their children with respect to 65 items on a 4-point rating scale (1 = not true; 2 = sometimes true; 3 = often true; and 4 = almost always true). Scores for five scales (social awareness, social cognition, social communication, social motivation, and restrictive and repetitive behavior) and a total score (SRS-T-Total) are calculated. This questionnaire is used for a dimensional diagnostic and severity assessment of symptoms of ASD. The majority of SRS items describe social communication behavior that is associated with autistic symptoms. Psychometric properties are reported to be excellent and the measures of diagnostic accuracy as a screening instrument for ASD are very high (e.g., Fombonne et al., 2012). The retest reliability ranges from adequate to very high (according to the classification of Strauss et al., 2006). The internal consistency of the SRS-Total Scale is high; the convergent validity with well-known tests is robust.

In the Quality of Life Inventory in Children and Adolescents (ILK, Mattejat and Remschmidt, 2006), the quality of life of the child is assessed by the parent’s proxy report in seven areas of daily life (with one question for each of the domains school, family, friends, alone, physical health, mental health, and overall) on a 5-point rating scale (1 = very good, 2 = rather good, 3 = partly, 4 = bad, and 5 = very bad). For these domains, the lower scores mean higher perceived quality of life of the child. Additionally, a Total Score can be calculated across all areas as LQ-Total-LQ0-28 (in this case the higher the score, the higher the reported quality of life). The retest reliability of the Quality of Life Inventory was found to be between marginal and high, and it is suitable and often used for the evaluation of psychotherapy.

There is an ongoing debate on the different approaches for measuring changes in self-report (e.g., Meyer et al., 2013). Direct measures may have the advantage of higher sensitivity to change. Therefore, we modified the answer format of the Quality of Life Inventory in Children and Adolescents to measure change of quality of life directly. The parent’s proxy report assessed whether the quality of life of their child improved or deteriorated compared to 8 weeks before on a 5-point rating scale (1 = very improved, 2 = somewhat improved, 3 = unchanged, 4 = somewhat deteriorated, 5 = very deteriorated) in the same domains as the original ILK version (Mattejat and Remschmidt, 2006). However, no psychometric characteristics are available for this new modified version.

The Parent Stress Questionnaire (ESF; Domsch and Lohaus, 2010) was developed to estimate parental life stress, role restriction, social support, and partnership. The stress level (Parental stress, ESF-PS) of the parents is assessed by 17 items asking about perceived parenting competencies (e.g., “I have doubts whether I am doing everything right in my upbringing”). Furthermore, the parents are asked in seven items about their perceived stress in the interaction with the child (e.g., “Sometimes I’m helpless about my child’s behavior”) and their daily parenting troubles (e.g., “I have to help my child with more daily things than I like“). The scale “role restriction” (ESF-RR) contains statements about perceived limitations associated with raising the child (e.g., “As a mother/father, I no longer have enough time for my hobbies”). The social support scale (ESF-SS) asks about support from the social environment. The internal consistency and retest reliability are adequate to very high (range of 0.76–0.92). For standardization, stanine values (1–9) were used. For parental stress and role restriction, high scores of stanine values (7–9) mean a clinically significant level. For the social support scale, low scores indicate a low level of perceived support.

The parenting questionnaire (EFB-K) is the German short-form adaptation of the Parenting Scale (PS; Arnold et al., 1993, German version by Naumann et al., 2010) that is a self-assessment scale of parenting behavior with 13 items. The endpoints describe effective or ineffective forms of certain parenting behavior in disciplinary situations, and the parents have to decide which kind of behavior they are more likely to come up with (appropriate or inappropriate parenting, e.g., “When my child behaves inappropriately, I shout at my child or I speak in a calm voice”). Each item is rated on a 7-point rating scale. A total score as well as two subscales of overreactivity and laxness can be analyzed.

### Measures

#### Primary Outcome Measures

The SRS is an instrument that is frequently used in autism-specific evaluation studies (e.g., Reichow et al., 2013; McConachie et al., 2015; Freitag et al., 2016). Therefore, we used the SRS as the primary outcome measure to measure social communication behavior of the child. In particular, T-scores of the scales Social Awareness (SRS-T-Awr), Social Cognition (SRS-T-Cog), Social Communication (SRS-T-Com), Social Motivation (SRS-T-Mot) and Restrictive and Repetitive Behavior (SRS-T-RRB), and the Total score (SRS-T-Total) of the Scale for Social Responsiveness (SRS; Constantino and Gruber, 2005; German translation: SRS; Bölte and Poustka, 2008) were calculated.

Also, the standardized Total Score of Quality of Life (Parent report: LQ-Total-LQ0-28) was used as a primary outcome for an overall measure of Quality of Life of the children. In addition, all seven domains were used for primary outcome analyses: Quality of life in school (QL-School), in relation to friends (QL-Friends), in relation to families (QL-Family), Quality of Life in relation to interests (QL-Alone), in relation to Physical Health (QL-Physical Health), in relation to Mental Health (QL-Mental Health), and Overall Quality of Life (QL-Overall).

#### Secondary Outcome Measures

As a secondary outcome measure, the Quality of Life Total Score-Change (QL-Change-Total Score) was used, together with all seven scores of the domains as described above (QL-Change-School; QL-Change-Friends; QL-Change-Family; QL-Change-Alone; QL-Change-PhysHeal; QL-Change-MentHeal; and QL-Change-Overall).

The scores of parental stress (ESF-PS), role restriction (ESF-RR), and social support (ESF-SS) were used as indicators for parents’ mental health and well-being.

For parenting behavior, we applied the total score of parenting (EFB-K-Total), the Overreactivity Scale (EFB-K-Overr), and the Laxness Scale (EFB-K-Lax).

### Statistical Analyses

For the post-assessments, group differences as changes to baseline were analyzed by means of one-way ANOVA. Effect sizes for group differences are reported in terms of standardized mean differences (SMD): Hedges’s *g*, rather than Cohen’s *d*, is used as an unbiased point estimator of effect sizes (Borenstein et al., 2009) because the former enables the computation of the 95% CI, also displayed in the forest plot of the systematic review of results.

For the follow-up-assessments, ANOVAs with three repeated measurements (baseline, post, and follow-up) were conducted. In cases violating the sphericity assumption (as checked by Mauchly’s test), the Greenhouse-Geisser correction was applied.

All statistical analyses were performed with SAS software, Version 9.4 (SAS Institute Inc., Cary, NC, United States). For hypothesis testing, a significance level of *α* = 0.05 was adopted. Concerning missing data, complete-case analyses were conducted, i.e., no imputation methods were applied.

## Results

The parents’ feedback at the end of the training was very positive. Altogether, 61 of 67 families (91.04%) completed the FETASS Parent Training. The dropout rate during the parent training was descriptively lower than in the TAU group, i.e., 6 out of 67 (8.96%) vs. 4 out of 33 (12.1%). At baseline, there were no significant differences between the two groups (FETASS vs. TAU) for all primary or secondary outcome measures, for age (range: 4;9–15;0), or for intellectual abilities (range: TAU: 67–136; FETASS: 72–133; see Table 2).

**Table 2:** Baseline sample characteristics for quantitative variables of chronological age, intellectual abilities, and social communication behavior, quality of life, parental stress, and parenting in the TAU and FETASS group.

	TAU			FETASS			*F*	*p*
	*n*	*M*	*SD*	*n*	*M*	*SD*
Age	29	10.04	2.11	28	10.52	2.53	<1	
IQ	29	98.79	15.43	28	99.43	14.04	<1	
SRS-T-Total	29	81.34	9.59	28	78.89	8.56	<1	
SRS-T-Awr	29	75.00	7.92	28	71.50	8.14	1.95	0.168
SRS-T-Cog	29	77.00	9.34	28	74.57	7.07	<1	
SRS-T-Com	29	82.79	12.68	28	80.71	11.39	<1	
SRS-T-Mot	29	75.82	9.90	28	76.46	10.17	<1	
SRS-T-RRB	29	79.90	11.47	28	78.71	10.61	<1	
QL-Total Score-LQ0-28	29	15.83	3.35	28	16.50	3.33	<1	
QL-School	28	2.93	1.36	27	2.59	0.89	<1	
QL-Family	29	2.21	0.90	27	2.41	1.01	<1	
QL-Friends	29	3.62	1.05	28	3.25	0.97	1.92	0.171
QL-Alone	29	2.48	1.30	27	2.30	1.03	<1	
QL-PhysHeal	29	2.03	0.73	28	2.11	0.92	<1	
QL-MentHeal	29	3.24	0.83	28	3.14	0.76	<1	
QL-Overall	29	2.69	0.81	28	2.68	0.61	<1	
ESF-PS	29	8.00	1.16	19	8.11	1.15	<1	
ESF-RR	29	6.90	1.59	19	7.53	1.43	1.95	0.170
ESF-SS	29	3.86	1.66	19	3.74	1.97	<1	
EFB-K-Total	29	3.01	0.82	22	3.23	1.02	1.58	0.215
EFB-K-Overr	29	3.51	1.05	22	3.97	1.26	3.37	0.074
EFB-K-Lax	22	2.57	0.90	22	2.57	1.28	<1	

TAU, treatment as usual; FETASS, Freiburg Parent Training; SRS, social responsiveness; SRS-T-Total, SRS total score; SRS-T-Awr, social awareness; SRS-T-Cog, social cognition; SRS-T-Com, social communication; SRS-T-Mot, social motivation; SRS-T-RRB, restricted interests and repetitive behavior; QL-Total-LQ0-28, quality of life total score; QL-School, quality of life in school; QL-Family, quality of life in families; QL-Friends, quality of life in relation to friends; QL-Alone, quality of life in relation to interests; QL-PhysHeal, quality of life in relation to physical health; QL-MentHeal, quality of life in relation to mental health; QL-Overall, overall quality of life; ESF-PS, parental stress; ESF-RR, role restriction; ESF-SS, social support of the parents; EFB-K-Total, parenting scale total score; EFB-K-Overr, overreactivity; EFB-K-Lax, laxness in parenting.

### Primary Outcome Measures

Concerning the primary outcome measures, no significant differences can be found after 8 weeks of FETASS intervention in comparison to TAU [SRS-T-Total: *F*(1, 54) = 0.01, *p* = 0.940, *g* = −0.02; QL-Total Score-LQ0-28: *F*(1, 55) = 0.01, *p* = 0.912, *g* = −0.03].

There are neither specific effects in favor of the FETASS intervention group concerning the different scales [SRS-T-Awr: *F*(1, 54) = 0.21, *p* = 0.647, *g* = −0.12; SRS-T-Cog: *F*(1, 54) = 0.21, *p* = 0.647, *g* = −0.12; SRS-T-Com: *F*(1, 54) = 0.28, *p* = 0.602, *g* = 0.14; SRS-T-Mot: *F*(1, 54) = 0.09, *p* = 0.765, *g* = −0.08; SRS-T-RRB: *F*(1, 54) = 0.43, *p* = 0.514, *g* = −0.17] nor specific short term effects concerning the domains of quality of life of the child [QL-School: *F*(1, 52) = 0.05, *p* = 0.825, *g* = −0.06; QL-Family: *F*(1, 54) = 0.13, *p* = 0.721, *g* = 0.09; QL-Friends: *F*(1, 55) = 0.37, *p* = 0.544, *g* = −0.16; QL-Alone: *F*(1, 52) = 0.03, *p* = 0.855, *g* = −0.05; QL-PhysHeal: *F*(1, 55) = 0.15, *p* = 0.701, *g* = −0.10; QL-MentHeal: *F*(1, 55) = 0.14, *p* = 0.708, *g* = 0.10; QL-Overall: *F*(1, 55) = 0.09, *p* = 0.765, *g* = 0.08].

**Figure 2:**
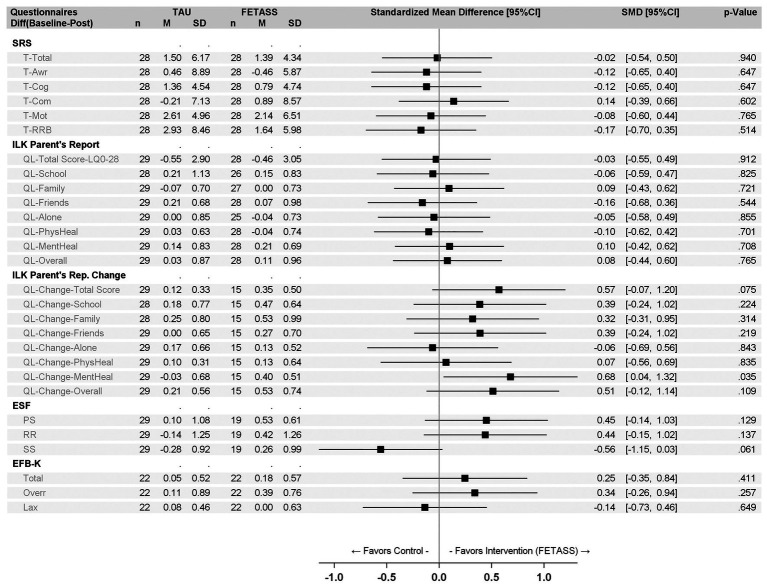
Forest plot with point estimates and 95% CI of standardized mean differences of the primary and secondary outcome measures (for abbreviations see Table 2).

Point estimates and confidence intervals of effect sizes for the primary outcome measures are part of the forest plot in Figure 2.

### Secondary Outcome Measures

In the Total Score of the quality of life (QL-Change-Total Score), no significant improvements could be found [*F*(1, 42) = 3.32, *p* = 0.075, *g* = 0.57]. Quality of life in relation to “Mental Health” of the child improves significantly in the FETASS group compared to the TAU group [QL-Change-Mental Health: *F*(1, 42) = 4.73, *p* = 0.035, *g* = 0.68] after training.

Improvements in parental stress and role restriction do not reach significance [ESF-PS: *F*(1, 46) = 2.39, *p* = 0.129, *g* = 0.45; ESF-RR: *F*(1, 46) = 2.29, *p* = 0.137, *g* = 0.44]. Descriptively, the social support of the parents tends to decrease in the FETASS group [ESF-SS: *F*(1, 46) = 3.70, *p* = 0.061, *g* = –0.56].

The parenting behavior scales (Total and Overreactivity) do not achieve any significance [EFB-K-Total: *F*(1, 42) = 0.69, *p* = 0.411, *g* = 0.25; EFB-K-Overr: *F*(1, 42) = 1.32, *p* = 0.257, *g* = 0.34]. The parenting scale “laxness” shows no changes at all in both groups [EFB-K-Lax: *F*(1, 42) = 0.21, *p* = 0.649, *g* = −0.14].

For the secondary outcome measures, point estimates and confidence intervals of effect sizes are also displayed in the forest plot in Figure 2.

### Long-Term Effects

For the primary outcome measures of communication behavior, a trend of improvement 6 months after training can be described, but all fall short of the adopted significance level [SRS-T-Total, *F*(2, 30) = 2.61, *p* = 0.090; see also Table 3]. For the follow-up measures of quality of life, there is a significant improvement [QL-Total Score-LQ0-28: *F*(2, 22) = 3.81, *p* = 0.038]. Also, the quality of life in the domain “Alone” [QL-Alone: *F*(1.333, 13.332) = 4.38, *p* = 0.047; Greenhouse-Geisser correction] shows significance, indicating an improvement in the child’s ability to organize his activities by himself. Further, a significant reduction of parental stress after 6 months is obtained [ESF-PS: *F*(2, 22) = 5.10, *p* = 0.015]. The comparison between the follow-up and the two time points (t1 + t2) is significant for a reduction of parental stress over time [*F*(1, 11) = 6.71, *p* = 0.025].

**Table 3:** Baseline (t1), post (t2), and follow-up (t3) statistics of the primary and secondary outcome measures (*N* = 16).

	Baseline (t1)		Post (t2)		Follow-up (t3)		*F*	*p*	Sign. *a priori* contrasts
	*M*	*SD*	*M*	*SD*	*M*	*SD*
SRS-T-Total	79.63	12.99	79.25	11.02	76.86	11.46	2.61	0.090	
SRS-T-Awr	72.13	13.29	71.25	9.27	68.69	8.72	1.70	0.207	
SRS-T-Cog	73.88	12.45	74.31	10.69	72.06	9.90	1.71	0.206	
SRS-T-Com	79.94	12.14	80.06	11.92	78.88	14.04	0.31	0.669	
SRS-T-Mot	73.88	14.70	74.13	14.08	71.38	16.20	1.62	0.220	
SRS-T-RRB	79.88	14.33	81.19	14.02	77.63	10.56	1.62	0.220	
QL-Total Score-LQ0-28^1^	16.83	2.52	17.08	2.68	19.42	2.68	3.81	0.038^*^	t3>(t1+t2)
QL-School^2^	2.80	0.79	3.00	0.82	2.60	1.07	0.92	0.399	
QL-Family^1^	2.17	0.83	2.08	0.90	2.00	0.603	0.19	0.793	
QL-Friends^1^	3.58	0.67	3.25	0.87	2.83	0.937	3.51	0.063	
QL-Alone^3^	2.36	1.21	2.45	1.03	1.72	0.90	4.38	0.047^*^	t3<(t1+t2)
QL-PhysHeal^1^	2.00	0.74	2.25	0.75	1.75	0.75	1.57	0.232	
QL-MentHeal^1^	2.92	0.67	2.83	1.11	2.42	0.792	2.29	0.127	
QL-Overall^1^	2.25	0.62	2.25	0.621	2.17	0.72	0.15	0.757	
ESF-PS^1^	7.58	1.51	7.25	1.86	6.75	2.05	5.10	0.015^*^	t3>(t1+t2)
ESF-RR^1^	7.25	1.29	7.83	1.40	6.75	1.96	2.88	0.088	
ESF-SS^1^	3.58	1.56	3.42	1.62	4.08	1.93	2.01	0.162	
EFB-K-Total^1^	2.90	0.82	2.77	0.77	2.78	0.81	0.32	0.733	
EFB-K-Overr^1^	3.18	1.03	3.06	0.99	2.85	0.97	0.80	0.463	
EFB-K-Lax^1^	2.72	0.89	2.65	0.76	2.88	1.09	0.97	0.395	

SRS, social responsiveness scale; SRS-T-Total, SRS-total score; SRS-T-Awr, SRS-social awareness; SRS-T-Cog, SRS-social cognition; SRS-T-Com, SRS-social communication; SRS-T-Mot, SRS-social motivation; SRS-T-RRB, SRS-restricted interests and repetitive behavior; QL, quality of life inventory; QL-Total-LQ0-28, quality of life total score; QL-School, quality of life in school; QL-Family, quality of life in families; QL-Friends, quality of life in relation to friends; QL-Alone, quality of life in relation to interests; QL-PhysHeal, quality of life in relation to physical health; QL-MentHeal, quality of life in relation to mental health; QL-Overall, overall quality of life; ESF, parenting stress questionnaire; ESF-PS, parental stress; ESF-RR, role restriction; ESF-SS, social support of the parents; Parenting Scale, EFB-K; EFB-K-Total, parenting scale total score; EFB-K-Overr, overreactivity; EFB-K-Lax, laxness in parenting.

* *p* < 0.05.

1 *N* = 12.

2 *N* = 10.

3 *N* = 11.

The parenting measures do not show any significant changes over time [EFB-K-Total: *F*(2, 22) = 0.32, *p* = 0.733; EFB-K-Overr: *F*(2, 22) = 0.80, *p* = 0.463; PS-Lax: *F*(2, 22) = 0.97, *p* = 0.395]. Descriptively, overreactivity shows a slight trend to further decrease after 6 months (see Table 3).

For the direct change measurement of quality of life (see Table 4), significant improvements can be described in the total score and the quality of life in relation to friends after 6 months [QL-Change-Total Score: *S* = 33; *p* = 0.017; QL-Change-Friends: *S* = 10.5; *p* = 0.031].

**Table 4:** Quality of life (change) at the 6-month follow-up (*N* = 14).

	Follow-up (t3)			
	*M*	*SD*	*S*	*p*
QL-Change-Total Score	0.31	0.42	33	0.017^*^
QL-Change-School	0.07	1.21	0.5	1.00
QL-Change-Family	0.36	0.63	10	0.125
QL-Change-Friends	0.43	0.51	10.5	0.031^*^
QL-Change-Alone	0.21	0.43	3	0.250
QL-Change-PhysHeal	0.29	0.61	3	0.250
QL-Change-MentHeal	0.43	0.65	13.5	0.070
QL-Change-Overall	0.36	0.74	12.50	0.180

Quality of Life Inventory-Change, QL-change; QL-Change-Total-Score, quality of life total score-change; QL-Change-School, quality of life in school-change; QL-Change-Family, quality of life in families-change; QL-Change-Friends, quality of life in relation to friends-change, QL-Change-Alone, quality of life in relation to interests-change; QL-Change-PhysHeal, quality of life in relation to physical health-change; QL-Change-MentHeal, quality of life in relation to mental health-change; QL-Change-Overall, overall quality of life-change.

* *p* < 0.05.

## Discussion

The present pilot study aimed to investigate the acceptability of the FETASS program, a specific modularized training program for parents of children with ASD aged from preschool to pre-adolescence. Social communication behavior, quality of life of the child, parental stress level, and parenting were preliminary evaluated in a case-control comparison immediately after training and, exploratively, in a follow-up. According to Smith et al. (2007), this study can be considered as a “Phase-Two Evaluation”: After manualization of the intervention, the acceptability of the manual has to be checked and a pilot case-control testing has to be conducted. In a next step, efficacy of the training must be investigated in a randomized controlled design.

### Acceptability

The parents’ feedback of the training intervention was positive and we had a low dropout rate during training. We interpret this as high acceptance of the program. In summary, the training appeared feasible in outpatient clinical procedures with a high acceptance from the parents. However, it should be critically noted that further important feasibility measures, e.g., qualitative assessments of outcome measures or clear criteria of acceptability according to Eldridge et al. (2016) were not collected in this study.

### Social Communication Behavior

In our pilot study, no significant improvements in social communication behavior were found after the completion of the FETASS training compared to the TAU group or after 6 months in the follow-up.

Autism spectrum is a neurodevelopmental condition affecting individuals during their whole lifespan. Improvement of social communication behavior in ASD is a lengthy process that depends on a wide range of factors, such as social motivation and social cognition of the child, as well as early interventions or family factors. For these reasons, we were not surprised that changes in the social responsiveness could not be found after the short time of an 8-week parent intervention. In Pickles et al. (2016), effects on social communication behavior were found in a 6-year follow-up, but not directly after the 13 months of PACT intervention (Green et al., 2010). According to these findings and our results, we conclude that a primary outcome measure of communicative behavior after a short time of intervention is not sensitive enough. Future RCT trial should take into consideration other parameters of efficacy and long-term effects in follow-up. Especially, a dimensional measure of social responsiveness like the SRS (Constantino and Gruber, 2005) can be critically taken into consideration as the primary outcome, although the German version of the SRS (Bölte and Poustka, 2008) is widely applied and often used to evaluate social training of children with ASD (e.g., Freitag et al., 2016).

Indeed, in the autism community, there is a discussion about the appropriateness of reducing autism to a medical condition and to apply deficit-based instruments to measure the efficacy of an intervention. Proponents of the neurodiversity approach claim that interventions should not be aimed to “cure” autistic symptoms but rather to enhance interactions and communication with other people (Milton, 2014).

Autism Spectrum Disorder can be seen as a cluster of strengths and weaknesses with the characteristic of high diversity. Children with ASD show a specific way to communicate and interact with other people. The behavior problems of children with ASD regularly arise in the interaction with their environment and with neurotypical people, e.g., in families. Often, parents have problems in understanding their autistic children and in reacting appropriately. This, in turn, can be stressful for the children with ASD, and in consequence, the children show more challenging behavior, e.g., aggressive behavior, but also less social communication behavior or less social motivation with social withdrawal and more repetitive behavior.

With reference to the “SPELL-framework” of the National Autistic Society, Milton (2014) suggests that autism-specific interventions should provide important principles such as “Structure”, “Positive”, “Empathy”, “Low-arousal”, and “Links”. The FETASS-program contains many aspects of this framework (e.g., teaching the parents to provide “structure” (Session 3), “positive parenting” (Session 1), or teaching parents how to provide an environment of “low arousal,” e.g., by preparing critical situations (Session 6 + 7; see in Supplementary Material).

For a future RCT trial, it will be crucial to find appropriate measurements to assess (1) social communication behavior of the child and quality life of the child, but as well, to measure (2) positive, empathic, and structuring parenting (3) and factors of an appropriate environment.

In a future study, it could be useful to add an assessment of dyadic parent-child interaction, such as in the recent study protocol by Green et al. (2018).

In addition, recent research focuses on a new instrument to measure changes in communication behavior for autism intervention evaluation (BOSCC, Grzadzinski et al., 2016), which unfortunately was not available as we started with the project.

### Quality of Life

Improvements in quality of life of the child (QL-Total) were not found immediately after training. When considering the parent’s change report, a significant effect was found in the mental health of the children after training compared to TAU. Furthermore, significant long-term effects were found for the quality of life in different domains [Alone (“able to organize activities by her/himself”), Friends, and Total Score]. In conclusion, these findings are promising to intensify research about potential effects on mental health of the child after parent training.

However, since mental health and well-being is a very broad concept with multiple definitions and different measurement approaches, the current findings have to be confirmed by using other validated measurements of emotional states or behavioral problems in autism.

For a future study, the use of a direct assessment of changes or an assessment of the quality of life of the parents could also be considered.

### Parental Stress

Concerning parental stress, we found no significant reduction after the training, but there was a significant reduction of parental stress level at follow-up. In gaining a better understanding of autistic behavior through training, parents seem to develop more appropriate skills to manage certain daily life situations and have a lower stress level. Even so, parents seem to need some time to implement the strategies they have learned. As a long-term effect, a lower stress level of parents might contribute to an enhancement of the child’s development (Keen et al., 2010; Schutte et al., 2018; Crowell et al., 2019).

Surprisingly, parents describe a trend to decreased social support in the FETASS group just after training, which is contrary to our hypothesis. However, descriptively, this tendency is inverted in the follow-up measure showing an improvement in social support compared to baseline. A tentative explanation could be that the parents needed some time to establish more supportive conditions and to learn about the social support networks for families with children with ASD.

### Parenting

No improvements in parenting like overreactivity and laxness were found.

In the Triple P evaluation for parents with ASD by Whittingham et al. (2009), effects in overreactivity and laxness of the parents right after the training were reported. However, positive effects decreased slightly over time.

In contrast, the results of the present study show no preliminary evidence toward a reduction of parents’ self-reported overreactivity after the FETASS or in follow-up, which is not in line with our hypothesis. A reduced overreactivity can be considered as one aspect of positive parenting. For further research, more appropriate measurement of positive, empathic, and structuring parenting has to be found (see above).

### Feedback on Training Materials and Minor Adjustments for Target Population

The materials used in the workbook appear primarily suitable for children of primary school age with no significant speech delay. Therefore, we recommend the application of the manual to parents of children with autism, who have an intellectual ability (IQ) of or above 70, without pronounced language impairment and within an age range from 5;11 to 12;11 years.

In summary, no improvements in social communication behavior or quality of life of the child after the FETASS training compared to TAU were found. However, there are some promising preliminary results for long-term follow-up, particularly regarding quality of life of the child as well as reduction of parental stress.

### Limitations

The study follows the criteria of a Phase-Two Evaluation study according to Smith et al. (2007). Therefore, the most important limitation is the non-randomized design and the small sample size. There might have been additional factors decreasing the specific effects of the training group. Concurrent factors like autism-specific therapy of the child, medication, and school assistance were not controlled for. This should be accounted for in further studies. A general problem in psychotherapy evaluation studies is that it is difficult to find an outcome measure (i) that can be easily blinded, (ii) that is not affected by subjective biases, and (iii) that has a high degree of sensitivity to change. Primary outcome measures of communication behavior and quality of life in the present study may contain categories that are too broad to detect improvements. McConachie et al. (2015) point out that suitable tools for detecting changes achieved through intervention studies of young children with ASD are scarce. Finally, the sample size has to be enlarged in order to detect both small to moderate effect sizes as well as long-term effects of the training.

## Conclusion and Future Directions

Although the etiology of autistic spectrum disorders is mostly attributed to genetic and neurodevelopmental factors, there seems to be growing evidence that parenting can influence aspects of the autism phenotype. In particular, positive parenting and an improved understanding of the child’s needs can help parents in supporting their autistic children. (Greenberg et al., 2006; Baker et al., 2010, 2011a,b; Mandy and Lai, 2016; Crowell et al., 2019).

The important role of family characteristics is also stressed in Karst and van Hecke (2012), who propose a new transactional intervention model, which includes the influence of parents’ characteristics on children with ASD. Karst and van Hecke (2012) point out that “most interventions for ASD are evaluated only in terms of child outcomes, ignoring parent, and family factors that may have an influence on both the immediate and long-term effects of therapy.”

Baker et al. (2011a) mention that children with autism, like most children, are responsive to their family environment. In this line, changing the environment and family condition in providing a positive and low-arousal environment may be able to modify the communication and interaction abilities of children with ASD. There is evidence that providing a specific family environment that is suitable to the needs of the autistic child could be one important factor in contributing to a more positive social and psychological outcome (Howlin and Magiati, 2017). This study is a first attempt to address these factors.

At present, there is an ongoing ethical discussion about purposes and measurements in intervention studies (Lord et al., 2005; Smith et al., 2007; Spence and Thurm, 2010; Milton, 2014; McConachie et al., 2015). For future directions, there should be a consensus about (1) what the interventions for autism are aiming for, and (2) what kind of measurements can be used for evaluation of autism intervention. In future, other important measurements of the parent-based intervention should additionally be considered. This could be the assessment of emotional problems or stress-related reactions of the child, or measurements of family characteristics (e.g., family communication style, dyadic parent-child interaction, coping style, parental mental health problems, or life quality of the parents).

In conclusion, the present pilot study shows high acceptability of the FETASS Parent Training with a low dropout rate during the training. Although no significant changes in social communication behavior were found, the initial results are encouraging to investigate efficacy of the FETASS Parent Training in a future RCT trial. Especially, the results of our pilot study emphasize the importance of including follow-up measurements.

## Data Availability Statement

The datasets presented in this article are not readily available because of confidentiality reasons. Requests to access the datasets should be directed to bettina.brehm@uniklinik-freiburg.de.

## Ethics Statement

The studies involving human participants were reviewed and approved by the Ethics Committee of the University of Freiburg. The patients/participants provided their written informed consent to participate in this study.

## Author Contributions

BB, MB, RR, and CF developed the study concept and designed the study with assistance of JS. BB and JS coordinated the study, recruited the participants, and completed the data collection. BB and RR planned the statistical analyses and RR carried out it with contributions of BB. All authors made contributions to the interpretation of the data. BB drafted the initial manuscript. All authors reviewed and revised the manuscript and approved the submission of the final manuscript.

### Disclaimer

BB, JS, CF, and MB are authors of the manualized FETASS program and receive royalties from Springer for the FETASS book.

### Conflict of Interest

The authors declare that the research was conducted in the absence of any commercial or financial relationships that could be construed as a potential conflict of interest.

## Supplementary Material

The Supplementary Material for this article can be found online at:

https://www.frontiersin.org/articles/10.3389/fpsyg.2021.604851/full#supplementary-material
