# Supplementary figures and images for: Preliminary Evaluation of the FETASS Training for Parents of Children With Autism Spectrum Disorder: A Pilot Study
Source: Front Psychol. 2021 Apr 30;12:604851. doi: 10.3389/fpsyg.2021.604851 (PMC8120893; doi:10.3389/fpsyg.2021.604851)

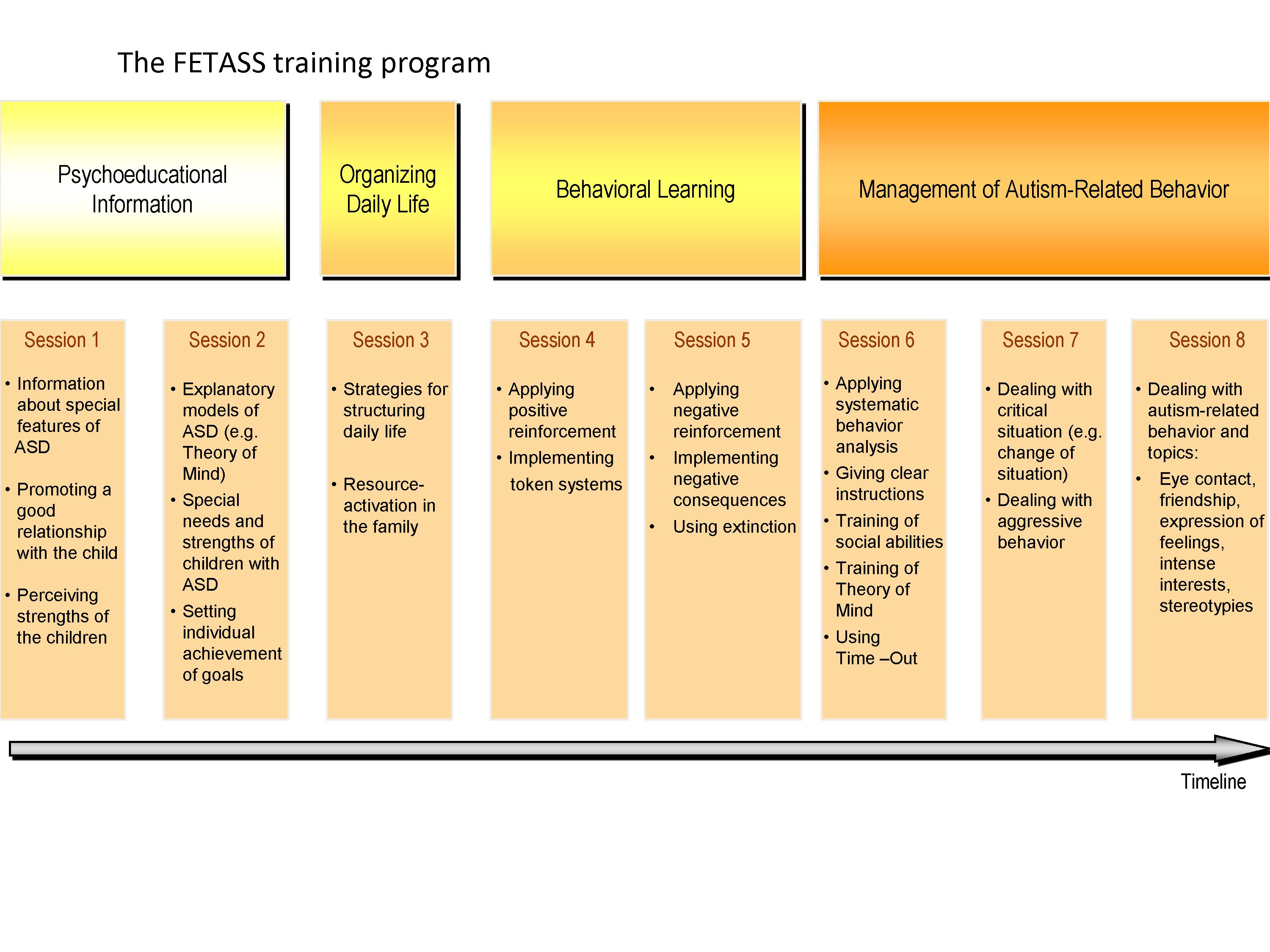

Supplement: Supplementary file 1 [file Image_1.TIFF]
